# Differential Myosin 5a splice variants in innervation of pelvic organs

**DOI:** 10.3389/fphys.2023.1304537

**Published:** 2023-12-12

**Authors:** Josephine A. Carew, Vivian Cristofaro, Raj K. Goyal, Maryrose P. Sullivan

**Affiliations:** ^1^ Urology Research, VA Boston Healthcare System, Boston, MA, United States; ^2^ Harvard Medical School, Boston, MA, United States; ^3^ Department of Medicine, Brigham and Women’s Hospital, Boston, MA, United States; ^4^ Department of Surgery, Brigham and Women’s Hospital, Boston, MA, United States

**Keywords:** protein splice variants, neurotransmission, myosin motor, peripheral nerve, pelvic organ

## Abstract

**Introduction:** Myosin proteins interact with filamentous actin and translate the chemical energy generated by ATP hydrolysis into a wide variety of mechanical functions in all cell types. The classic function of conventional myosins is mediation of muscle contraction, but myosins also participate in processes as diverse as exocytosis/endocytosis, membrane remodeling, and cytokinesis. Myosin 5a (Myo5a) is an unconventional motor protein well-suited to the processive transport of diverse molecular cargo within cells and interactions with multiprotein membrane complexes that facilitate exocytosis. Myo5a includes a region consisting of six small alternative exons which can undergo differential splicing. Neurons and skin melanocytes express characteristic splice variants of Myo5a, which are specialized for transport processes unique to those cell types. But less is known about the expression of Myo5a splice variants in other tissues, their cargos and interactive partners, and their regulation.

**Methods:** In visceral organs, neurotransmission-induced contraction or relaxation of smooth muscle is mediated by Myo5a. Axons within urogenital organs and distal colon of rodents arise from cell bodies located in the major pelvic ganglion (MPG). However, in contrast to urogenital organs, the distal colon also contains soma of the enteric nervous system. Therefore, the rodent pelvic organs provide an opportunity to compare the expression of Myo5a splice variants, not only in different tissues innervated by the pelvic nerves, but also in different subcellular compartments of those nerves. This study examines the expression and distribution of Myo5a splice variants in the MPG, compared to the bladder, corpus cavernosum of the penis (CCP) and distal colon using immunohistochemistry and mRNA analyses.

**Results/discussion:** We report detection of characteristic Myo5a variants in these tissues, with bladder and CCP displaying a similar variant pattern but one which differed from that of distal colon. In all three organs, Myo5a variants were distinct compared to the MPG, implying segregation of one variant within nerve soma and its exclusion from axons. The expression of distinct Myo5a variant arrays is likely to be adaptive, and to underlie specific functions fulfilled by Myo5a in those particular locations.

## 1 Introduction

Myosin 5a (Myo5a) is a dimeric motor protein that processively transports molecular cargos along actin filaments. The *Myo5a* gene is expressed by many secretory cell types (Desnos et al., 2003; Eichler et al., 2006; Conte et al., 2016) including the neurons/nerves of the central, enteric, and peripheral nervous systems (Prekeris and Terrian, 1997; Buttow et al., 2006; Trybus, 2008; Bridgman, 2009). The highly polarized morphology of neuronal cells, with functionally distinct extensions (dendrites and axon) protruding from the cell body (soma), each of which contains a complement of macromolecules and membranous organelles, implies the existence of mechanisms to sort and deliver the appropriate contents to each subcellular compartment. The longer-range transport of cargos within nerves depends on microtubules, which provide the tracks for cargo transport. The opposite polarity of most microtubules in dendrites and axons, which mediate interactions with different classes of motor proteins, provides one mechanism for cargo segregation. Dynein motors move cargo towards the microtubule minus-end (that is, into the dendrites) while kinesin motors move cargo towards the microtubule plus-end (into the axon) (Kardon and Vale, 2009; Hirokawa et al., 2010; Kapitein and Hoogenraad, 2011; Kneussel and Wagner, 2013). Once within the correct subcellular compartment, cargo can dissociate from dynein/kinesin motors and undergo short-range transport to its ultimate destination along actin filaments via interactions with myosin motors, including Myo5a. In addition to a transport function, Myo5a is also thought to serve as a tether that restricts the motion of cargo after it has been delivered to the correct location (Desnos et al., 2007).

The neural cargos of Myo5a are known to include organelles such as endoplasmic reticulum, multiprotein complexes including transcriptionally repressed mRNAs and ribosomes, and synaptic vesicles (Prekeris and Terrian, 1997; Wagner et al., 2011; McCaffrey and Lindsay, 2012; Calliari et al., 2014; Canclini et al., 2020). However, relatively little is known regarding regulation of the interactions between Myo5a and such diverse cargo which mediate their transportation to appropriate destinations, especially in peripheral nerves. Myo5a—cargo interactions have been primarily studied in brain and skin, the organs most obviously affected by mutation of the *Myo5a* gene (Wu et al., 1998; Wagner et al., 2011). In mice, ablation of the *Myo5a* gene expression results in the autosomal recessive dilute-lethal phenotype; that is, affected mice display both reduced pigmentation and neurological abnormalities which culminate in death by 3 weeks of age (Mercer et al., 1991). Similar lethal defects have been associated with null inheritance of the *Myo5a* gene in other species, including rat (Futaki et al., 2000; Landrock et al., 2018), dog (Christen et al., 2021) and horse (Brooks et al., 2010). Defective expression of the *MYO5a* gene in humans causes the autonomic recessive disorder, Griscelli syndrome Type I, which is characterized in affected infants by silvery grey hair color and signs of severe neurological impairment such as developmental delays, seizures, opisthotonus, and hypotonia (Griscelli et al., 1978; Griscelli and Prunieras, 1978). Interestingly, genetic changes in *Myo5a/MYO5a* genes that apparently affect only pigmentation have also been described in both mice and humans; these include mutation within, or even deletion of, a single exon of the Myo5a monomer (Huang et al., 1998; Menasche et al., 2003; Yilmaz et al., 2014).

Myo5a protein has a domain structure reflective of its biological functions. In the N-terminal segment, which includes the motor and lever arm, there are binding regions for ATP, actin, and the calcium-binding protein, calmodulin. The C-terminal segment contains coiled-coil regions that are required for dimerization, and a globular tail domain (GTD) which participates in cargo binding ((Trybus, 2008) and references therein). The actin binding and ATP-hydrolyzing domains of Myo5a are highly conserved to those of other myosin protein superfamily members. However, the lever arm of Myo5a is relatively long, and the presence of a coiled-coil region in the medial tail indicates dimerization of Myo5a monomers. Both of these characteristics predict that the step length of Myo5a with each cycle of ATP hydrolysis will also be relatively long, 36 nm, or one helical turn of the actin filament. Consequently, Myo5a dimers can move smoothly and efficiently along the filament surface ((Trybus, 2008) and references therein). Myo5a dimers are active, that is, capable of locomotion, only when stimulated by elevated Ca++ (Li et al., 2004) or when bound to molecular cargo (Thirumurugan et al., 2006); at other times they assume a folded conformation unable to hydrolyze ATP. Both the active and inactive forms bind to actin (Li et al.; Liu et al., 2006).

Partially overlapping with the coiled-coil domain, and just upstream of the GTD, is the Myo5a alternative exon segment. This region is comprised of six exons (sequentially termed A, B, C, D, E, and F) which can be spliced together in multiple arrangements without disturbing the reading frame (Mercer et al., 1991; Seperack et al., 1995; Huang et al.; Lambert et al., 1998a). Exons A, C and E are thought to be present in all variants, while exons B, D and F may or may not be included. In brain Myo5a, the exon arrangement ABCE predominates, while in skin and other tissues such as spleen, variants with other exon arrangements are expressed (Seperack et al., 1995). In patients with Griscelli syndrome who have abnormal pigmentation but no apparent neurological defects, alternative exon F is deleted (Menasche et al., 2003; Yilmaz et al., 2014). This exon is involved in efficient transport of pigment-containing granules (melanosomes) to the tips of skin melanocytes for their transfer to keratinocytes (Au and Huang, 2002). Binding of exon F to the accessory protein, melanophilin, bridges the interaction between Myo5a and melanosomes by concomitantly binding to Rab27a, a protein associated with melanosome membranes (Fukuda et al., 2002).

The Myo5a splice variants expressed in peripheral tissues other than skin melanocytes, have not yet been extensively characterized. We have recently identified splice variants expressed by stomach and bladder using PCR methodologies (Carew et al., 2021; Carew et al., 2022). In all stomach sections (antrum, fundus, body and pylorus), the exon arrangement identified in brain Myo5a, ABCE, was detected along with other arrangements such as ACDE, ACEF, and ACDEF. Although the latter variants were also detected in bladder, the ABCE variant was not. In the stomach, the enteric nervous system is embedded between the longitudinal and circular muscle layers (the myenteric plexus) and between the circular muscle and the mucosa (the submucosal plexus). These enteric plexuses, containing the soma and dendrites of the enteric nervous system, are inextricable from the muscle tissue penetrated by the axons. The rodent bladder, however, is innervated only by the axons of nerves whose soma/dendrites reside at a distance, within the MPG (Drengk et al., 2000; Keast, 2006). These anatomical differences between stomach and bladder suggested that Myo5a variant mRNAs may be compartmentalized within peripheral nerves, with the ABCE variant confined to soma/dendrites while other variants are restricted to axons. Further, Myo5a mRNA was recovered from isolated bladder, which contains only the severed axons of pelvic nerves. These data confirm reports from other groups that Myo5a mRNA, as well as Myo5a protein bound to kinesins, undergoes transport into the axon (McCaffrey and Lindsay, 2012; Calliari et al., 2014; Canclini et al., 2020) and presumably can be translated within this compartment.

In rodents, the MPG innervates not only bladder, but also a number of other pelvic tissues. The work presented here analyzes the Myo5a variants present in other such tissues—the CCP, distal colon, and MPG itself, as well as the bladder. The distal colon is innervated extrinsically by the MPG, and also (like the stomach) by the enteric plexuses. Since presumably all Myo5a transcription and mRNA splicing takes place in the nucleus of the expressing cell, we hypothesized that all pelvic nerve Myo5a variants, including ABCE, would be detectable in MPG and distal colon. However, we expected that a subset of the total variant transcripts, excluding ABCE, would be detectable in CCP and bladder, since those organs contain only the axons of nerves emanating from the MPG.

## 2 Materials and methods

### 2.1 Animals

All animal procedures were approved by the Institutional Animal Care and Use Committee of the VA Boston Healthcare System. Male C57/BL6 (RRID:IMSR_JAX:000664) mice were obtained from Jackson Labs (Bar Harbor, ME, United States). Between 8 and 14 weeks of age, animals were euthanized by CO_2_ asphyxiation, and organs were quickly dissected and either stabilized in RNA-Later solution (R0901, Sigma-Aldrich, St Louis, MO, United States), or embedded in OCT compound, immediately snap frozen and stored at −80°C.

### 2.2 Confocal Microscopy

Full thickness sections of urinary bladder, distal colon, and CCP were cut on a cryostat (12–16 µm) and fixed with cold acetone for 10 min. Whole mount preparations from dissected major pelvic ganglia were also fixed as described. After rinsing in phosphate buffered saline (PBS), tissue preparations were blocked and permeabilized with 5% donkey serum and 0.05% triton X-100 in PBS for an hour. For double labelling, tissues were incubated overnight at 4°C with rabbit primary antibody against Myo5a (Novus NBP1-92156, RRID:AB_11017070; 1:300 dilution) along with an antibody against a neuronal marker: either mouse anti-NeuN (Millipore, MAB377; RRID:AB_ 2313673), mouse anti-ßIII tubulin (Abcam, ab7751, RRID:AB_306045) or mouse anti-synaptophysin (Abcam, ab8049, RRID:AB_2198854). After extensive washing with PBS, tissues were incubated for 2 h at room temperature with an affinity-purified goat anti-rabbit secondary antibody coupled to AlexaFluor-568 (Thermo-Fisher Scientific: A21206, RRID:AB_2535792) followed by an AlexaFluor 488 conjugated donkey anti-mouse secondary antibody (Thermo-Fisher Scientific: A11031, RRID:AB_144696). In separate sections processed in parallel, the primary antibodies were omitted from the procedure to control for nonspecific binding of secondary antibodies.

Tissue sections were mounted using Fluoromount-G (0100–01, Southern Biotech, Birmingham, AL, United States) and examined by confocal microscopy (Zeiss, LSM 710) using a ×40 water objective. For image acquisition of double-labelled tissue, slides were scanned separately at each excitation wavelength to minimize emission crosstalk. Images were obtained from maximum-density projections of 8–10 optical slices.

### 2.3 mRNA extraction, cDNA preparation and PCR analyses

Three independent samples of each pelvic tissue (MPG, bladder, CCP, distal colon) plus one positive control (brain), were dissected from each of three animals and processed for total RNA. RNAs from MPG and brain were purified with the Qiagen RNeasy Plus Mini kit (28104); RNAs from pelvic tissues were purified using the RNeasy Fibrous Tissue Mini kit (74704). Tissues were disrupted in the kits’ lysis buffer, RLT, supplemented with ß-mercaptoethanol (M3148, Sigma-Aldrich) using the Tissue Lyser II mill-bead apparatus (Qiagen, Gaithersburg, MD, United States) by two cycles of 2 minutes each at 30Hz with a 5 mm stainless steel bead (69989, Qiagen). RNA concentration and purity were determined by measurement of absorbances at 260nm and 280 nm on the Nanodrop 1000 UV-vis spectrophotometer (Thermo-Fisher Scientific) using the RNA nucleic acid quantification program. An A260/A280 ratio of ∼2.0 for each sample indicated adequate purity for downstream applications.

**FIGURE 1:**
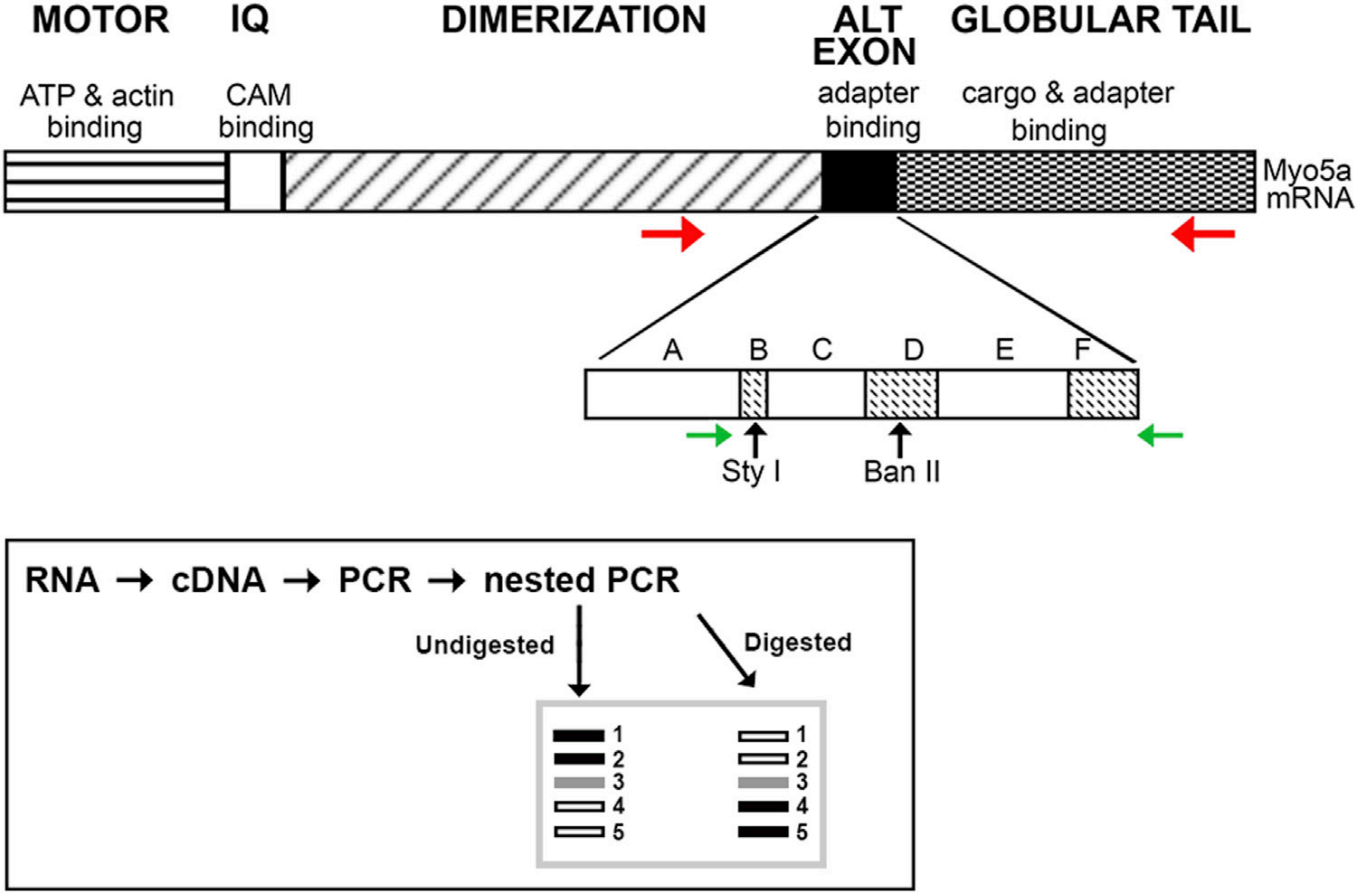
Schematic of Myo5a. The regions of Myo5a encoding the ATP binding, motor, calmodulin-binding (IQ), alternative exon and globular tail domains, and the known functions of each, are portrayed at top. The expansion depicts organization of the alternative exon domain; exons A, C and E are constitutively expressed in all variants, whereas exons B, D and F are optional. Horizontal arrows represent positions of PCR primers (red, initial reaction; green, nested reaction) while approximate positions of unique Sty I and Ban II restriction enzyme sites within exons B and D, respectively, are indicated by vertical arrows. The inset depicts the PCR and restriction enzyme methodology used to assess the presence and proportion of Myo5a splice variants.

To prepare total RNA for PCRs, 100 ng were converted to cDNA in a final volume of 20 μL using the Superscript IV VILO mastermix (11756050, Thermo-Fisher Scientific). Aliquots of the cDNA (1 μL) were used as templates for 50 μL initial PCR reactions. Initial reaction employed a forward primer upstream of Myo5a exon A (Figure 1) and reverse primer within the GTD (primer pair 1; forward, 5′ GCA​GAA​ACT​GAA​GAC​ATT​GCA​CC 3′ and reverse, 5′ ATT​CTG​GCG​AGA​CGT​GTT​GT 3′). Products were purified with the Qia-quick PCR-purification kit (28104, Qiagen). A 1 μL aliquot of each initial purified PCR was then used as a template for nested PCR with internal primer pair 2 (forward, 5′ GTC​CTC​ATC​TTG​AGG​TCG​CA 3′ and reverse, 5′ATG​TTG​ACT​GGC​CGG​ATA​GG 3′). All primers were obtained from Thermo-Fisher Scientific. For both initial and nested PCR, twenty-five cycles were performed under standard conditions (denaturation at 94°C, annealing at 58°C, and extension at 72°C) in an Eppendorf thermal cycler (Hauppauge, NY, United States) using the Taq PCR mastermix (201445, Qiagen). Initial PCR reactions included a no-template control (NTC), containing full reaction mixture—buffer, TAQ polymerase, primers, dNTPs—but no cDNA as template for amplification. A 1 μL aliquot of this NTC reaction was also used as a template in the control reaction with the nested primers.

A schematic illustrating this workflow is included in Figure 1 (inset). Except for the 5’ nested primer, which is within exon A (present in all variants), all primers hybridize to sequences completely outside of the alternative exon region. In particular, the reverse primer for nested PCR described here differs from that used in our previous analyses, being located within the GTD. Since the entire region potentially carrying the alternative exons B, D, and/or F was amplified, the nested PCR products obtained represent both the configurations and the relative proportions of Myo5a variant transcripts expressed by each tissue.

Aliquots of the nested PCR products were digested with excess restriction enzyme (20–40 units of either Sty I, R3500 or Ban II, R0119) at 37°C for 2–16 h in Cut-Smart reaction buffer. All reagents were from New England Biolabs (Ipswich, MA, United States). Undigested and digested fragments were assessed in comparison to mass standards (15628019, Thermo-Fisher Scientific) by electrophoresis in Tris/acetate/EDTA buffer (9868, Ambion/Thermo-Fisher Scientific), on 2% MetaPhor agarose (50180, Lonza, Rockland, ME United States) gels. Gels were prepared just before use, according to the manufacturer’s protocol, and contained the fluorescent intercalating dye, Sybr-green (S33102, Thermo-Fisher Scientific).

Gels were imaged using an Amersham Imager 600 (General Electric, Pittsburgh, PA, United States). For every lane in Figure 4, the fluorescence intensity of each band was measured and corrected for fragment size. The fluorescent signal present in each individual band was expressed as a proportion of the total fluorescence intensity, that is, the sum of all band intensities, in that lane. The proportional values for each position in the replicates from a given tissue were then averaged and plotted +/- SE. The same process was followed for analysis of restriction digestion (Figures 5, 6), except that fluorescence intensity was measured at the positions corresponding to the undigested and the digested bands, in all lanes derived from each sample. Statistical significance was determined by using the Student’s t-test to compare the proportional intensity of each band under undigested *versus* digested conditions. p values below 0.05 were considered significant.

## 3 Results

### 3.1 Myo5a protein is expressed in pelvic and enteric nerves

**FIGURE 2:**
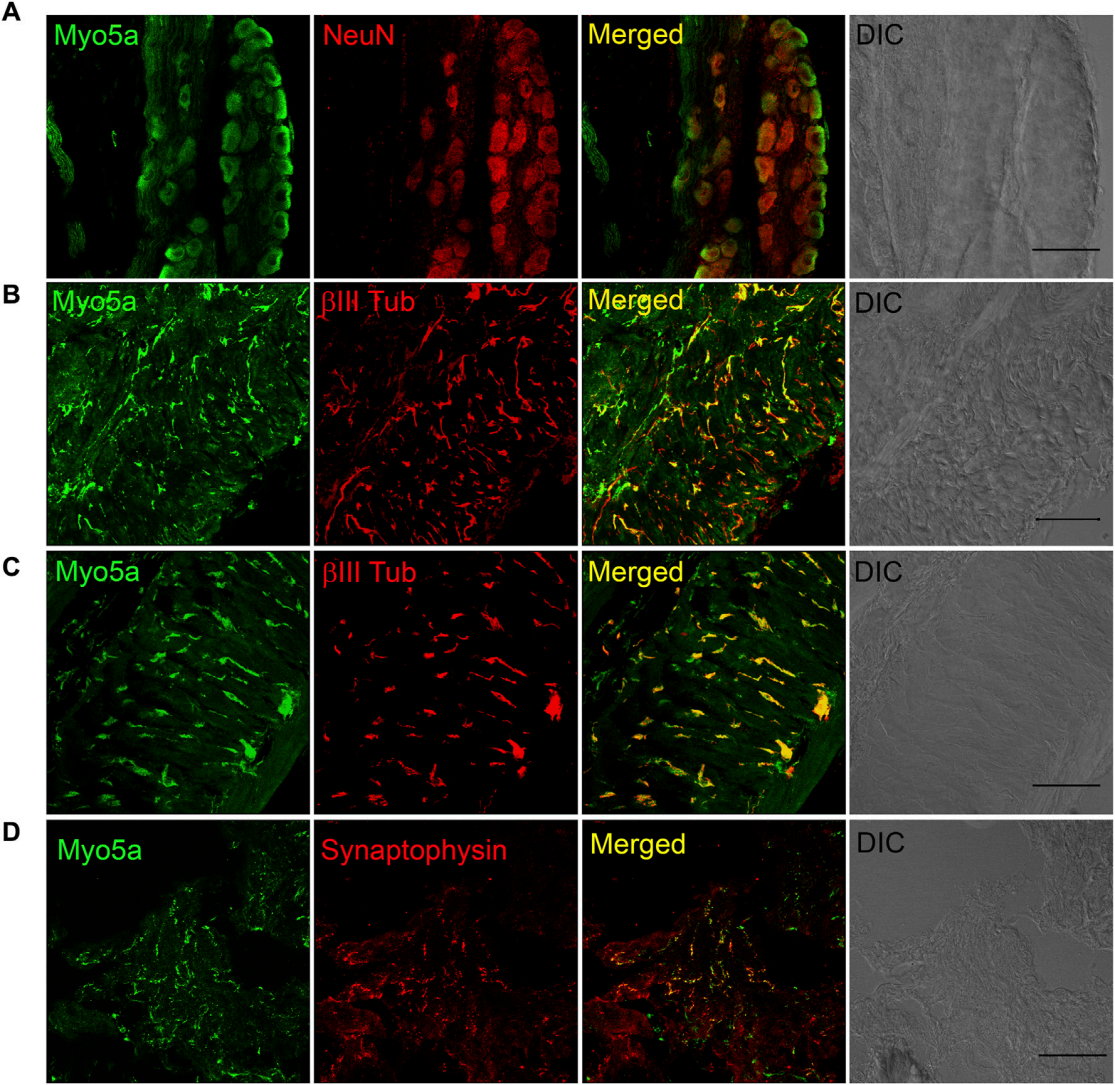
Distribution of Myosin 5a (Myo5a) in different murine organs innervated by the pelvic plexus. **(A)** Whole-mount preparation of the MPG displays abundant staining for Myo5a which was extensively co-localized with the neuronal marker NeuN. Myo5a immunoreactivity was also detectable on fibers running throughout the bladder **(B)** and the distal colon **(C)** Co-localization of Myo5a signal with β-III tubulin confirmed the neuronal nature of myo5a distribution in these tissues. **(D)** Myo5a immunoreactivity was abundantly detected and co-localized with the synaptic-vesicle marker synaptophysin in the CCP. Tissue topography was shown in the differential interference contrast (DIC) image from each preparation. Magnification ×40; scale bars = 50 μm.

Myo5a immunoreactivity was detected in whole mount preparations of the MPG. Double labeling with the neural marker NeuN showed that Myo5a was localized in cell bodies. Myo5a was also detected in neurites within the MPG (Figure 2A). Antibodies against Myo5a yielded abundant immunoreactivity throughout the tissues from the urinary bladder (Figure 2B), the distal colon (Figure 2C) and the CCP (Figure 2D). In double labelled preparations, Myo5a staining was extensively colocalized with immunoreactivity generated by neural markers β-III tubulin or synaptophysin.

### 3.2 Multiple splice variants of Myo5a are identified in pelvic tissues by nested PCR

Discriminating among Myo5a protein variants by Western blotting is not possible due to two factors: the lack of commercial antibodies recognizing the variant exons, and the small differences in protein mass predicted for the different Myo5a splice variants. The alternative exons B, D and F encode 3, 27 and 25 amino acids respectively relative to a protein backbone of 1828 amino acids for full-length protein lacking all three alternative exons. Therefore, the Myo5a splice variants expressed in the various pelvic tissues were identified at the mRNA level by PCR.

**FIGURE 3:**
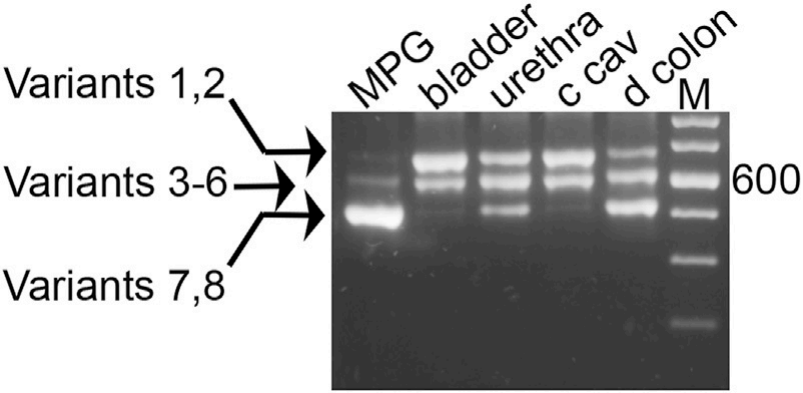
Myo5a PCR product sizes of pelvic tissues. Nested PCR was performed with a representative sample from each of four tissues, and compared by 2% agarose gel electrophoresis. An intervening lane was removed (white line). Three distinct bands were observed per sample, and in comparison to the DNA size markers (M, 100 bp ladder, with weighted 600 bp standard) they were of approximate mass 700, 600, and 500 bp. The arrows at left indicate the possible variants corresponding to each band, from Table 1.

As shown in Figure 3, nested PCR produced three product bands separable by electrophoresis, in a representative sample from each pelvic tissue. The pattern of the distal colon included easily detectable amounts of all three product bands, and stood in strong contrast to the arrays seen for MPG (mostly the smallest band, with traces of the larger products), and for bladder and CCP (ample amounts of the two larger PCR bands with a trace of the smallest). Whether these trace bands give rise to functional Myo5a variants is not known. Further analysis was concentrated on the more abundant forms expressed in each tissue.

As mentioned above, fragments very close in size cannot be resolved by this technique alone, so each band potentially contains multiple species closely related in mass. Theoretically, eight different variants, as shown in Table 1, could be generated by the nested PCR. In comparison to the mass standards in far right lane of Figure 3, the observed bands generated from the pelvic tissues are approximately 700 bp (and could be composed of variant species 1—2 from Table 1), 600 bp (variant species 3–6) and 500 bp (variant species 7–8). The uppermost band is large enough to contain sequence encoding all three variable exons: B (9 bp), D (81 bp) and F (75 bp). The central band has a mass consistent with presence of either exons B+D, D only, B+F, or F only. The smallest band might or might not include exon B, but is not large enough to include either exons D or F.

**TABLE 1:** Potential splice variants of the Myo5a gene.

Species	Exons	Size (BP)	Sty I DIGEST (BP)	Ban II Digest (BP)
Variant 1	ABCDEF	677	50 and 627	209 and 468
Variant 2	A_CDEF	668	no cleavage	200 and 468
Variant 3	ABCDE_	602	50 and 552	209 and 393
Variant 4	A_CDE_	593	no cleavage	200 and 393
Variant 5	ABC_EF	596	50 and 546	no cleavage
Variant 6	A_C_EF	587	no cleavage	no cleavage
Variant 7	ABC_E_	521	50 and 471	no cleavage
Variant 8	A_C_E_	512	no cleavage	no cleavage

The predicted sizes of the undigested, Sty I digested, and Ban II digested fragments resulting from nested PCR, of each theoretically possible murine Myo5a splice variant, are shown. Each species gives a unique, identifiable signature of fragment sizes prior to and following restriction enzyme cleavage.

**FIGURE 4:**
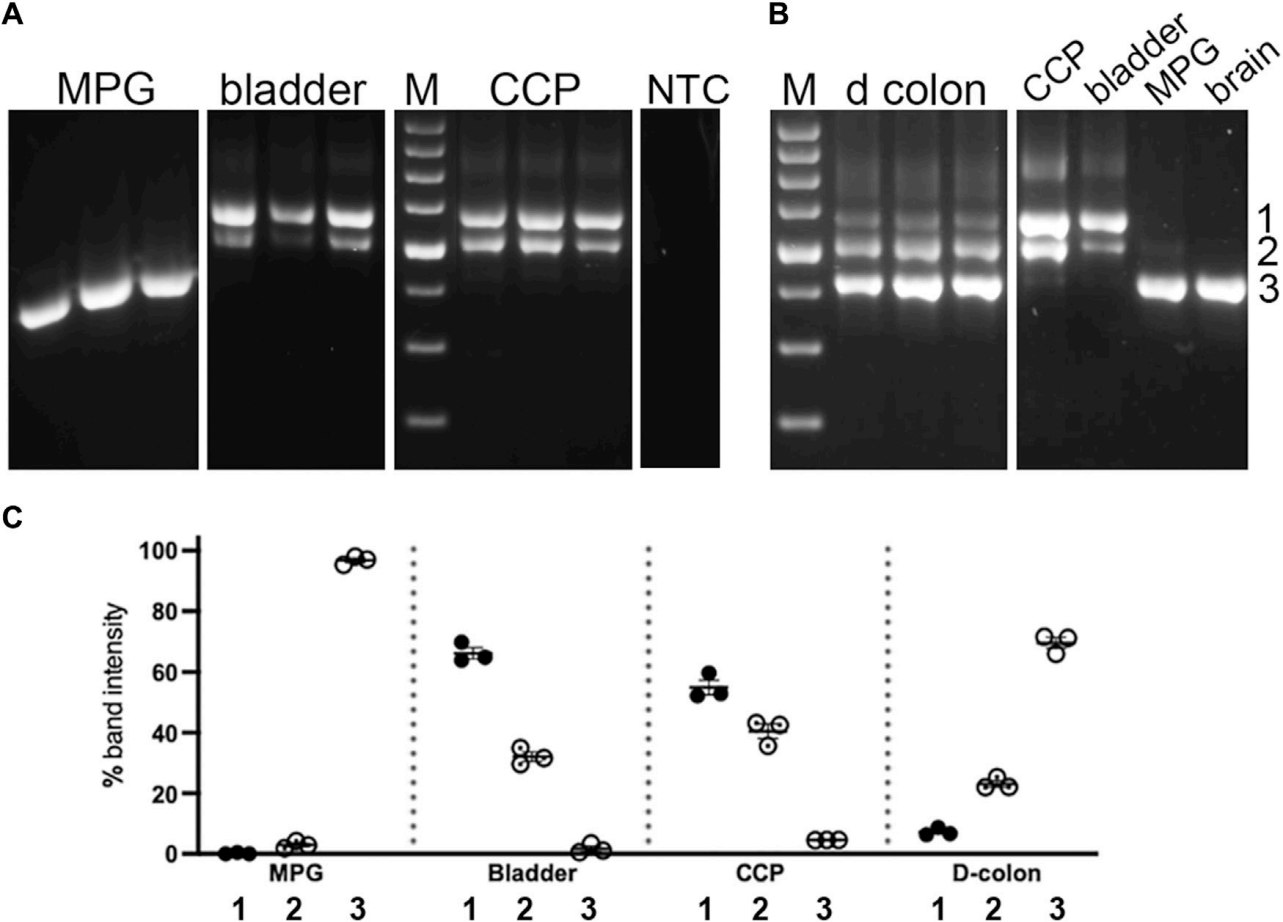
Undigested PCR products of pelvic tissues. Nested PCR was performed on three replicates of each tissue type, which were analyzed by 2% agarose gel electrophoresis, Panel **(A)** shows replicates from MPG, bladder and CCP. In parallel with experimental reactions, a control reaction which included all PCR reaction components except cDNA, was run. An aliquot of this reaction was then included as a template in a control nested PCR reaction. No PCR product was detected in this reaction (NTC). Panel **(B)** shows replicates from distal colon, as well as a single sample each from CCP, bladder, MPG and brain for comparison with the samples from panel **(A)**. For both panels, white lines indicate the removal of intervening lanes from the images. Standards differing by 100 bp increments (with weighted 600 bp standard) were run on each gel (M). Numbers 1 through 3 at the far right indicate band identities. **(C)** Relative percentages of these three PCR products were determined, then replicates were average and graphed +/- SEM. ∼700 bp band 1, filled circles; ∼600 bp band 2, dotted circles; ∼500 bp band 3, open circles.

The abundance of these three product sizes were next compared in replicate undigested samples from nested PCR of the pelvic tissues. The bands were quantitated, and their relative proportions in each sample were determined and graphed, as shown in Figure 4. Although the control PCR reaction did not generate a discernable product, all nested PCR reactions run with tissue sample cDNAs gave rise to an array of product sizes characteristic of each tissue. Again, the largest product predominated in bladder and CCP, where it comprised ∼50–60% of the total. This product was barely detectable (∼5%) in distal colon and MPG. The central product could be easily discerned in bladder, CCP, and colon (20%–45%) but not in MPG (∼5%). Finally, the smallest product was found to predominate in MPG and distal colon, but to be nearly undetectable in CCP and bladder. The product distribution of bladder was mirrored by that of CCP, but both were different from distal colon and MPG. Also, distal colon and MPG showed different distribution patterns compared to one another.

### 3.3 Myo5a exon B is recognized only in MPG and distal colon

**FIGURE 5:**
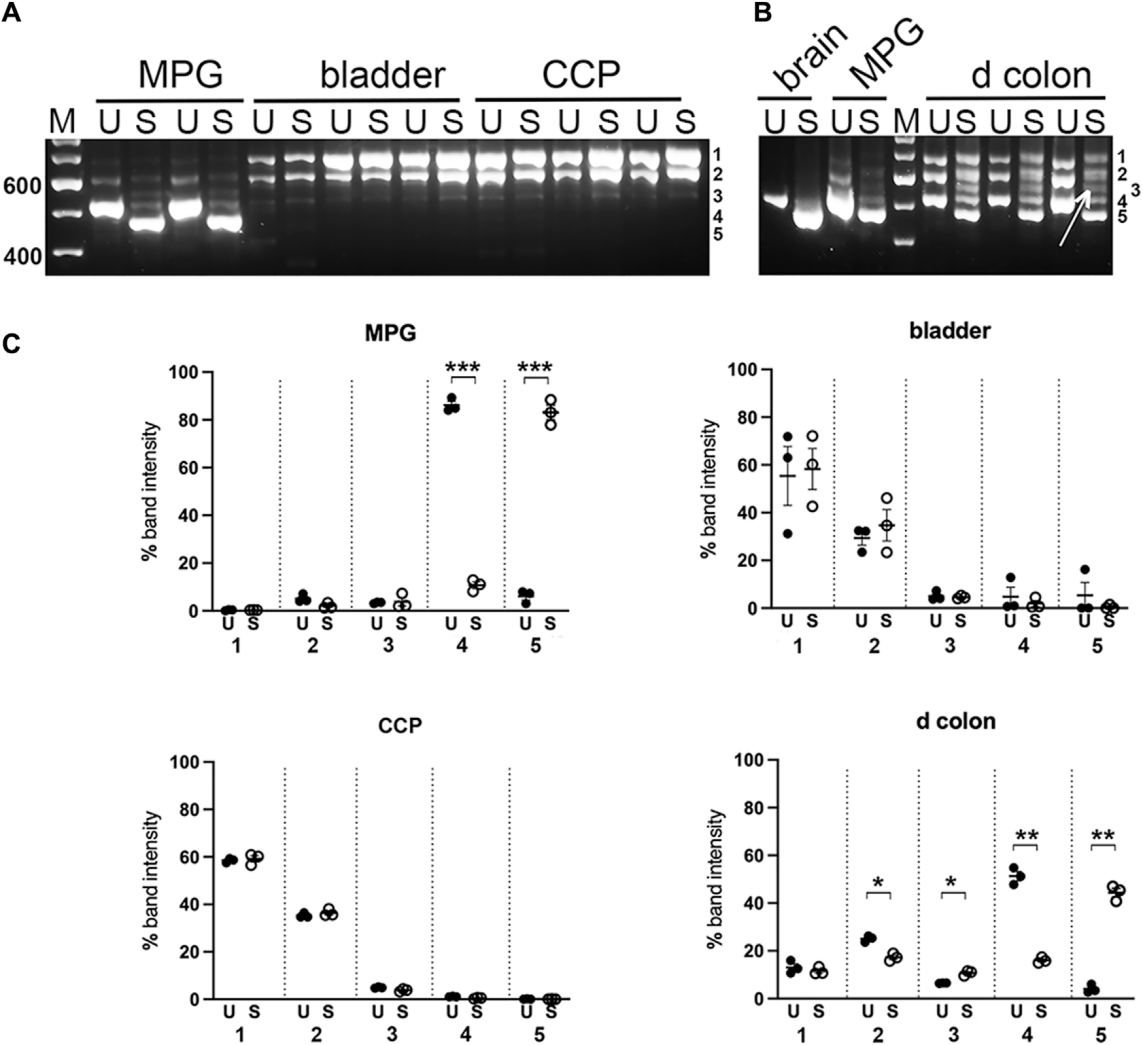
Sty I digestion of PCR products from pelvic tissues. Undigested (U) and Sty I digested (S) nested PCR pairs from replicates of MPG, bladder, CCP **(A)**, or from distal colon (d colon) and MPG, with brain for reference **(B)** were electrophoresed and analyzed as described in Methods. For mass estimation, a 100 bp DNA ladder with weighted 600 bp standard was run (M). Numbers 1 through 5 at the right of each image correspond to band identities. Bands 1, 2 and 4 correspond to the upper (∼700 bp), middle (∼600 bp), and lower (∼500 bp) bands present in undigested samples and remaining undigested by Sty I. Band 3, indicated by arrow in panel B, represents a Sty I cleavage product of band 2; band 5 represents a cleavage product of band 4. **(C)**, Fluorescence was quantitated at each of the five band positions and converted to the percentage of total fluorescence recovered in that lane. Replicates at each band position were averaged and graphed +/- SEM, (U = undigested, closed circles; S = Sty I digestion, open circles). *n* = 3 per tissue. Significant changes in fluorescence at each band position due to Sty I digestion are indicated (*p < 0.05; **p < 0.01; ***p < 0.001).

To further distinguish among fragments of similar mass, the nested PCR products were digested separately with restriction enzymes Sty I and Ban II, to indicate the presence of exon B or D, respectively, as indicated in Figure 1; Table 1. The undigested and digested samples were then analyzed as described above. Quantitative comparisons for abundance of the undigested *versus* Sty I—digested bands are shown in Figure 5. By inspection of the various undigested *versus* digested counterparts in the gels of panels A and B, evidence for inclusion of exon B was found most clearly in MPG and distal colon. In MPG the Sty I digestion of the smallest PCR product appeared to be complete, indicating that exon B was present exclusively in the variant 7 arrangement described in Table 1, ABCE. However, in distal colon there was only partial Sty I digestion of this size product, suggesting that the variant 8 species, ACE, was also present along with ABCE. In addition, a Sty I digested fragment of ∼550 bp was also observed in colon (see arrow in Figures 5B). Because Sty I cleavage removes 50 bp from the 5’ end of a nested PCR product spanning exon B, these data indicated that the ∼600 bp PCR product in distal colon included either or both variant 3, ABCDE, and variant 5, ABCEF. Aside from this, there was no suggestion of exon B in the larger PCR product pair from any tissue.

### 3.4 Myo5a exons D and F are identified in pelvic tissues

**FIGURE 6:**
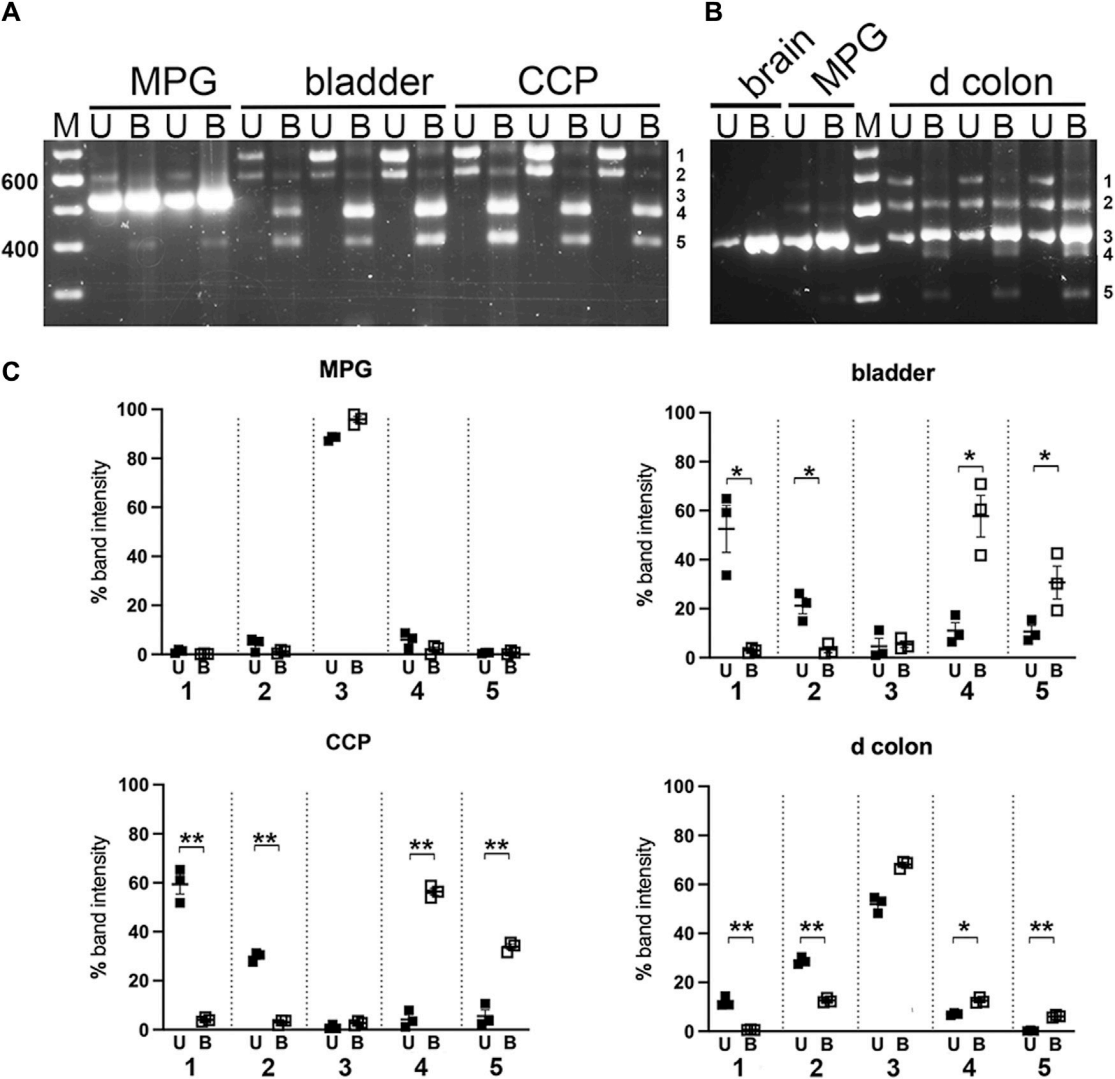
Ban II digestion of PCR products from pelvic tissues. Undigested (U) and Ban II digested **(B)** nested PCR pairs from replicates of MPG, bladder, CCP **(A)**, or from distal colon with MPG and brain for reference **(B)** were electrophoresed and analyzed as described in Methods. For mass estimation, a 100 bp DNA ladder with weighted 600 bp standard was run (M). Numbers 1 through 5 at the right of each image correspond to band identities. Bands 1, 2 and 3 correspond to the upper (∼700 bp), middle (∼600 bp), and lower (∼500 bp) bands present in undigested samples and remaining undigested by Ban II. Band 4 represents a cleavage product of band 1; band 5 represents a cleavage product of band 2. **(C)** Fluorescence was quantitated at each of the five band positions and expressed as a percentage of total fluorescence recovered in that lane. Replicates at each band position were averaged and graphed +/- SEM. (U = undigested, closed squares; B = Ban II digestion, open squares). *n* = 3 per tissue. Significant changes in fluorescence at each band position due to Ban II digestion are indicated (*p < 0.05; **p < 0.01).

Digestions of the nested PCR products with restriction enzyme Ban II are shown in Figure 6; this enzyme is expected to remove 200/209 bp from the 5’ end of the PCR product if exon D is included. As anticipated, Ban II did not cleave the smallest PCR product from MPG or colon. In contrast, the pair of larger PCR products from bladder, CCP and colon were extensively digested by Ban II, indicating that variant 2, ACDEF, (generating the 468 bp digestion product) and variant 4 arrangement described in Table 1, ACDE, (generating the 393 bp digestion product) were the major species found in bladder and CCP. The Ban II digestion pattern for distal colon, however, differed in that very little of the middle PCR product could be cleaved, suggesting that the unexpected variant 6, ACEF, and/or variant 5, ABCEF, (due to partial digestion of this sized fragment with Sty I, see above) were expressed in this tissue only.

## 4 Discussion

This work analyzed the Myo5a variants present at the mRNA level in several representative pelvic tissues. Accordingly, two tissues of the rodent genitourinary tract with innervation from the MPG (bladder and CCP of the penis) as well as the MPG itself, were used for PCR and restriction enzyme analysis in comparison to the distal colon, which is innervated both by the enteric nervous system and the MPG. The ABCE variant of Myo5a is so abundantly expressed in neurons of the central nervous system that it is referred to as the brain form and might be expected to be the most prevalent form in the peripheral nervous system as well. However, our previous work implied that this was not the case: the ABCE variant is detectable in stomach sections but undetectable in bladder, while variants containing the alternative exons D and F were identified in both tissues (Carew et al., 2021; Carew et al., 2022). Based on immunohistochemistry data, Myo5a protein is found predominantly in nerve trunks and extensions where it colocalizes with axonal markers such as synaptophysin and β-III-tubulin. Therefore, we expected mRNA expression of Myo5a to be limited largely to peripheral nerves in these tissues. An anatomical feature distinguishing innervation of stomach from that of bladder, is the presence of plexuses housing the soma/dendrites of enteric nerves in the stomach. Rodent bladder, in contrast, is innervated by the axons of peripheral nerves whose soma/dendrites reside in the MPG. Taken together, these data suggested that within enteric and peripheral nerves, expression of the Myo5a ABCE variant might be restricted to the soma/dendrites, while any other Myo5a variants expressed might be restricted to the axons.

The data presented here support this contention. The nested PCR product corresponding to the brain variant, ABCE, is predicted to be 521 bp and to be cleaved by the restriction enzyme Sty I to fragments of 471 and 50 bp. The only tissue investigated which expressed a preponderance of an ∼500 bp PCR fragment completely digested by Sty I, was the isolated MPG. Aside from MPG, only the distal colon (which included the enteric plexuses) gave rise to a significant amount of an ∼500 bp PCR product. Unexpectedly however, this product band was partially refractory to Sty I, suggesting that the ACE variant was included. Also surprisingly, in distal colon unlike bladder and CCP, the ∼600 bp PCR product was partially cleaved by Sty I. This indicated that either or both the ABCDE/ABCEF variants were expressed in this tissue. No Sty I digestion fragments were detected in digests of either bladder or CCP PCR products, indicating these tissues were devoid of variants containing exon B.

When the PCR products were next digested with Ban II to identify variants including exon D, the largest product band (∼700 bp) was fully cleaved in all tissues in which this species could be discerned (bladder, CCP, and distal colon), generating a digestion product of 468 bp. Based on the mass, resistance to Sty I, and susceptibility to Ban II, these data indicate that the ∼700 bp PCR product corresponded exclusively to ACDEF. This species was the major variant of Myo5a detected in bladder and CCP. Similarly, the presence of exon D was indicated in nearly all the ∼600 bp PCR product of bladder and CCP based on the degree of Ban II digestion observed, and thus corresponded to variant ACDE in these tissues. In the distal colon, however, the ∼600 bp PCR product was also partially resistant to Ban II. Coupled with its partial resistance to Sty I, these data signaled expression of a mixture of variants such as ABCDE, ACDE, ABCEF and ACEF were expressed in this tissue only, among those analyzed.

Since *in situ* hybridization had indicated that Myo5a mRNA is primarily localized to nerve soma/dendrites within the myenteric plexus (Carew et al., 2021), and immunohistochemistry indicated extensive colocalization with neural markers (such as β-III-tubulin, synaptophysin or NeuN) (Carew et al., 2021; Carew et al., 2022), Myo5a expression in CCP, distal colon and MPG, as well as bladder, was expected to be largely restricted to nerves. But because variant-specific anti-Myo5a antibodies are unavailable, it was not possible to assign expression of a particular mRNA variant detected to a particular location by immunohistochemistry. This is a potential drawback of our study; however expression of the ABCE, or brain variant, can be confidently assigned to the soma, accounting for its detection in the distal colon as well as the MPG. The other forms detected, such as ACDEF and ACDE, which predominate in CCP and bladder, thus must be present in the axons. Interestingly, the presence of trace amounts of the larger nested PCR products in MPG might indicate the presence of these Myo5a variants in the axons within this tissue.

Previous work indicates that a complex array of Myo5a variants can be expressed even by a single cell. For example, it has been demonstrated that mRNAs corresponding to the full-length ABCDEF, the intermediate-length ABCDE and/or ABCEF and the short-length ABCE variants could be detected in cultured fibroblasts, keratinocytes, and melanocytes, as well as in mixed leukocytes isolated from blood; whereas only in melanocytes were variants ACDEF and ACE also found (Lambert et al., 1998b; Westbroek et al., 2003). In melanocytes, functional differences were noted for the different Myo5a variants. For example, the recombinant ABCE protein did not interact with melanosomes, whereas recombinant ABCEF and ABCDEF did. While it was expected that only Myo5a variants with exon F would mediate melanosome transport (Lambert et al., 1998b; Fukuda et al., 2002) it was further shown that ABCDEF and ABCEF were not equally capable of melanosome association, despite both including exon F. Instead, the presence of exon D diminished the ability of exon F to mediate this interaction, implying that even closely-related variants may fulfill subtly different roles within a single cell type (Westbroek et al., 2003).

Our analysis indicated that variant ACEF was not abundantly expressed in pelvic tissues, although its presence can be inferred in distal colon by a degree of resistance of the ∼600 bp fragment to Ban II digestion, and its presence was inferred as a minor species in bladder (Carew et al., 2022). In contrast, the ∼600 bp fragment from bladder and CCP was extensively cleaved by Ban II, suggesting it was comprised mostly of ACDE in these tissues. In distal colon only, variants such as ABCDE/ABCEF, and ACE, were detected.

Interestingly, each of the nucleated cell types investigated by Lambert et al. expressed easily discernable amounts of the Myo5a brain variant, ABCE, which we detected only in tissues containing peripheral nerve soma. Taken together, these data imply that in all Myo5a—expressing secretory cells, this particular variant protein is likely to be restricted to the cell’s nuclear and perinuclear regions. In our study, it was clear that the soma/dendrites of the nerves serving the bladder and CCP, which are located in the MPG, demonstrated near-exclusive expression of the ABCE variant. Only trace amounts of the larger variants could be seen in nested PCR from MPG, as might be expected if those variants were differentially sorted and rapidly exported to axons following their transcription.

The highly polarized neuron must confront an important issue: how to maintain over time the appropriate protein content of its elaborate extensions, despite their distance from the soma which contains the nucleus and is the site of gene transcription. In peripheral axons, this problem is particularly acute, since the extensive distance between the soma and the distal axon may exceed the capacity of protein transport processes to resupply macromolecular constituents critical for local functions, within a reasonable timeframe [reviewed in (Roy, 2014; Black, 2016)]. Although transport of a particular protein and of the mRNA encoding it into extensions are not mutually exclusive processes, the capacity to carry out local protein synthesis gives axons the flexibility to finely regulate the level and activity of proteins required for specialized axonal functions both spatially and temporally [reviewed in (Giuditta et al., 2008; Sahoo et al., 2018; Mofatteh, 2020)].

There is now much evidence that the mRNAs to be transported into axons initially interact with various RNA binding proteins (usually through sequence-specific interactions involving particular motifs in their untranslated regions, or UTRs). In these ribonucleoprotein complexes, or RNPs, the mRNA is held in an inactivated state (Kar et al., 2018). The RNPs undergo long-range active transport along microtubules in axons mediated by kinesin motor proteins, then short-range transport along actin filaments mediated by myosin motor proteins, in order to reach their destinations (Kalinski et al., 2015). The mRNAs themselves thus participate in the regulation of gene expression, as a consequence of their presence in the axon and their translation in response to an appropriate signal from the external milieu.

In a polarized cell such as a differentiated neuron, the transport of specific mRNAs into cellular extensions and their local translation into proteins confer a number of advantages, compared to the transport of centrally translated proteins. For example, sorting of mRNAs using sequences in their 3′UTRs or other untranslated regions is efficient, since a single RNA binding protein is capable of interaction with multiple mRNAs which may include very different coding sequences (Holt and Schuman, 2013). Further, since a single mRNA can be translated into multiple copies of its encoded protein, local control of translation permits rapid adjustment of the local proteome in response to a local stimulus (Buxbaum et al., 2015). Also, the protein translated on-demand could carry different post-translational modifications than the centrally translated protein, or be required to fulfill a unique functional role at the distal site, as is the case for ß-actin in the developing axon (Liao et al., 2015). The nascent protein, translated on-demand would also have a longer functional half-life in the axon, while its unnecessary presence in other subcellular compartments of the nerve would simultaneously be precluded.

It is not known whether locally-synthesized Myo5a fulfills specialized functions in the peripheral nerve axon, distinct from functions it might have in the other compartments. However, Myo5a is thought to play important roles in transport of synaptic vesicles within varicosities and their retention near active zones until a depolarization event, both critical functions for optimal regulation of neurotransmitter release. Our previous work characterized impairments of both inhibitory and excitatory neurotransmission in the fundus, CCP and bladder of the dilute-brown-nonagouti, or DBA, mouse, a strain carrying a defect that reduces the splicing of exon D and exons D+F in Myo5a transcripts (Chaudhury et al., 2014; Carew et al., 2021; Carew et al., 2022). It is tempting to speculate that these impairments arose from the inability of Myo5a lacking exons D and F to carry out essential neurotransmission functions in peripheral nerve axons.

## Data Availability

The original contributions presented in the study are included in the article/Supplementary Materials, further inquiries can be directed to the corresponding author.
